# Simple Self-Assessment Tool to Predict Osteoporosis in Taiwanese Men

**DOI:** 10.3389/fmed.2021.713535

**Published:** 2021-11-16

**Authors:** Dung-Huan Liu, Chung-Yuan Hsu, Pei-Ching Wu, Ying-Chou Chen, You-Yin Chen, Jia-Feng Chen, Shan-Fu Yu, Tien-Tsai Cheng

**Affiliations:** ^1^Department of Physical Medicine and Rehabilitation, China Medical University Hospital, Taichung, Taiwan; ^2^Doctoral Degree Program of Biomedical Science and Engineering, College of Biological Science and Technology, National Yang Ming Chiao Tung University, Hsinchu, Taiwan; ^3^Department of Physical Therapy, Graduate Institute of Rehabilitation Science, China Medical University, Taichung, Taiwan; ^4^Division of Rheumatology, Allergy and Immunology, Department of Internal Medicine, Kaohsiung Chang Gung Memorial Hospital, Chang Gung University College of Medicine, Kaohsiung, Taiwan; ^5^Department of Chinese Medicine, China Medical University Hospital, Taichung, Taiwan; ^6^Department of Biomedical Engineering, National Yang Ming Chiao Tung University, Taipei, Taiwan; ^7^The Ph.D. Program for Neural Regenerative Medicine, College of Medical Science and Technology, Taipei Medical University, Taipei, Taiwan

**Keywords:** osteoporosis, self-assessment tool, men, Taiwan, fracture

## Abstract

**Background:** Although the self-assessment tools for predicting osteoporosis are convenient for clinicians, they are not commonly used among men. We developed the Male Osteoporosis Self-Assessment Tool for Taiwan (MOSTAi) to identify the patients at risk of osteoporosis.

**Methods:** All the participants completed a questionnaire on the clinical risk factors for the fracture risk assessment tool. The risk index was calculated by the multivariate regression model through the item reduction method. The receiver operating characteristic (ROC) curve was used to analyze its sensitivity and specificity, and MOSTAi was developed and validated.

**Results:** A total of 2,290 men participated in the bone mineral density (BMD) survey. We chose a model that considered two variables (age and weight). The area under the curve (AUC) of the model was 0.700. The formula for the MOSTAi index is as follows: 0.3 × (weight in kilograms) – 0.1 × (years). We chose 11 as the appropriate cut-off value for the MOSTAi index to identify the subjects at the risk of osteoporosis.

**Conclusions:** The MOSTAi is a simple, intuitive, and country-specific tool that can predict the risk of osteoporosis in Taiwanese men. Due to different demographic characteristics, each region of the world can develop its own model to identify patients with osteoporosis more effectively.

## Introduction

Due to the aging population, osteoporosis in men is becoming a global health problem.

Aging men lose ~1% of their bone mineral density (BMD) each year while 20% of men over the age of 50 will develop osteoporosis-related fractures in their lifetime (1). In Taiwan, awareness and policy interventions have stabilized the epidemic trend of osteoporosis (2). The proportion of the population over 50 years of age is expected to increase from 32% (7.5 million) in 2013 to 57% (11.9 million) in 2050. The life expectancy of men will increase from 80 years in 2013 to 83 years in 2050 (3). The Taiwanese men have a higher annual hip fracture rate (4). Additionally, 20.67% of these men died within 1 year of injury (5). This percentage was much higher than the percentage of women diagnosed with hip fractures. Therefore, identifying these men is essential to prevent osteoporosis-related fractures.

According to the WHO classification criteria, dual-energy X-ray absorptiometry (DXA) is the gold standard diagnostic test for osteoporosis (T-score, −2.5). Due to the limited supply of DXA machines and restrictions on the reimbursement of medical expenses in some of the rural areas, the people living in these areas are not able to receive the routine DXA and BMD scans. To improve the relevance and effectiveness of these scans, several tools have been developed, such as Osteoporosis Self-Assessment Tool for Asians (OSTA) and the National Osteoporosis Foundation recommendations in 2013 (NOF 2013) (6, 7). Since 1993, several studies have associated osteoporosis with aging and low body weight (8, 9). In 2001, Koh et al. proposed OSTA as a convenient method to identify this risk due to weight and age (7). NOF 2013 determines the high-risk patients belonging to different age groups based on the number of clinical risk factors. In addition, this tool is used to identify the individuals who should be DXA tested. Currently, OSTA is validated in men of various races but is not directly evaluated in Taiwanese men yet. Additionally, the efficacy of OSTA is not directly compared with NOF 2013. Therefore, this study developed a risk index called Male Osteoporosis Self-Assessment Tool for Taiwan (MOSTAi) and validated it by using a separate cohort and comparing it with NOF 2013.

## Materials and Methods

### Participants

Between 2008 and 2011, the Taiwan Osteoporosis Association implemented a tour program for the entire island to evaluate BMD. This plan included a trained nurse, a DXA machine (Explorer; Hologic Inc., Waltham, MA, USA), a bus from the International Society for Clinical Densitometry, and a radiographer. The buses can be used in various locations on request and the DXA machines are used for measuring BMD. There is a total of 104 stations in Taiwan.

In order to be included as a part of this study, the patients had to meet the following inclusion criteria: men who had informed consent and were aged 50 years and older. The patients were excluded from the study if their hips were previously fractured or replaced. All the included men were randomly assigned a 1:1 development or validation cohort to form the MOSTAi index. The local Institutional Review Board of Chang Gung Memorial Hospital (102-1878B), Taoyuan City, Taiwan, approved the study. All the participants provided written informed consent for this study.

### Data Collection and Measurements

A trained nurse interviewed each participant and a questionnaire was filled out to be accessed by fracture risk assessment tool (FRAX). The clinical risk factors such as weight, height, use of glucocorticoids, previous fractures, rheumatoid arthritis (RA), secondary osteoporosis, gender, age, parental hip fracture, smoking, and drinking were assessed. The BMD measurements were performed in both the hips and the lumbar spine of all the patients using a DXA machine inside the bus. The least significant changes (LSCs) in each area were 3.93% (total hip), 4.19% (femoral neck), and 3.17% (lumbar spine) (10). The BMD value is based on the reference value of Asian young women aged 20–29 years (11).

The participants are divided into the non-osteoporosis or osteoporosis risk groups that require BMD testing based on whether they meet one of the following criteria in the NOF 2013 recommendation. The criteria include women of 65 years of age and older, or postmenopausal women younger than 65, who have one or more clinical risk factors for fractures, such as low weight, previous fractures, use of low bone mass, or high-risk medication use related to low bone mass, or a disease or condition related to bone loss. The criteria also evaluated the diagnostic performance of NOF 2013 in predicting osteoporosis in the population we studied.

### Statistical Analysis

The *t* and the chi-square tests analyzed the continuous and categorical variables, respectively. FRAX assessed the possible risk factors for the model development. A univariate analysis was included in the multiple variable logistic regression models after identifying the statistically significant risk factors (*p* < 0.05). The next step was to constitute the MOSTAi index through the multiple variable logistic regression analyses and item reduction methods that were based on the major risk factors.

The WHO criteria define osteoporosis with a *T*-score of ≤ −2.5 at any site (lumbar spine, femoral neck, or total hip) (12). A receiver operating characteristic (ROC) curve analysis was performed to assess the ability of MOSTAi to distinguish between the subjects diagnosed with and without osteoporosis. The sensitivity was defined as the proportion of men diagnosed with osteoporosis (*T*-scores ≤ −2.5) who had 1 tested positive (i.e., index values below the cut-off), and the specificity was defined as the proportion of men diagnosed without osteoporosis who tested normal (i.e., having index values above or equal to the cutoff). The area under the curve (AUC) compared the performance of MOSTAi with OSTA and NOF 2013. In order to test the statistical difference between the AUCs, Delong's test was performed; for the sensitivity and specificity, Mcnemar's test was performed. All the analyses were performed using the SPSS statistics. A *P*-value of <0.05 indicated a statistically significant difference.

## Results

**Figure 1:**
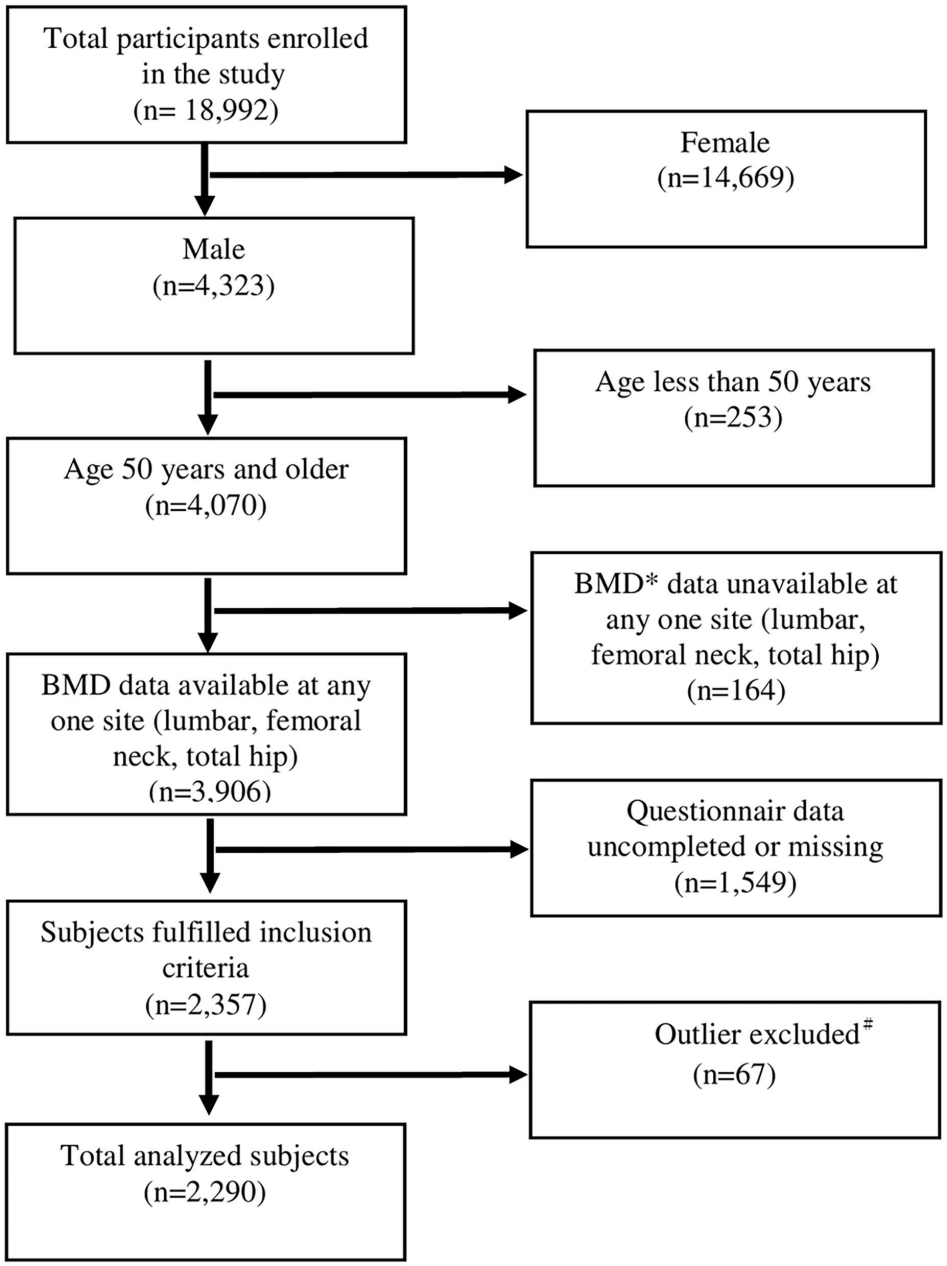
Inclusion of the participants in the study. ^*^Participants whose BMD was missing at any one site for any reasons were excluded. ^#^Participants with extreme values (deviating from the mean by more than three times the standard deviation), including demographics and BMD were excluded for data analysis.

The Taiwan OsteoPorosis Survey (TOPS) recruited a total of 18,992 participants, including 4,323 men (22.8%) and 14,669 women (77.2%). The analyses excluded the patients having inaccessible BMD data, incomplete or missing questionnaire data based on FRAX, and age group < 50. The distribution of these participants is shown in Figure 1. A total of 2,290 men were evaluated and randomly assigned a development (*n* = 1,145) or validation (*n* = 1,145) cohort.

Table 1 shows the characteristics of the development and validation cohorts. The average age is 69.6 ± 9.6 years. The average body weight and average body mass index (BMI) were 65.9 ± 9.8 kg and 24.1 ± 3.2 kg/m^2^, respectively. For all the characteristics and clinical risk factors, there were no significant differences between the development and validation cohorts (*p* > 0.05).

**Table 1:** The demographic characteristics of all the participants.

**Characteristics**	**Total number *n* = 2,290(100%)**	**Development Cohort *N* = 1,145 (50%)**	**Validation Cohort *N* = 1,145 (50%)**	***P*-value**
Age (years)	69.6 ± 9.6	69.9 ± 9.5	69.4 ± 9.6	0.294
Height (cm)	165.4 ± 6.1	165.5 ± 6.0	165.2 ± 6.1	0.323
Weight (kg)	65.9 ± 9.8	65.9 ± 10.1	65.8 ± 9.6	0.727
Body mass index (kg/m^2^)	24.1 ± 3.2	24.0 ± 3.3	24.1 ± 3.1	0.868
**BMD (g/cm** ^**2**^ **)(T-score)**				
Lumbar spine (g/cm^2^)	1.01 ± 0.15	1.02 ± 0.15	1.01 ± 0.15	0.950
(T-score)^*^	−0.7 ± 1.4	−0.7 ± 1.4	−0.7 ± 1.4	
Femoral neck (g/cm^2^)	0.76 ± 0.13	0.76 ± 0.13	0.76 ± 0.13	0.777
(T-score)^*^	−1.2 ± 1.0	−1.2 ± 1.0	−1.3 ± 1.0	
Total hip (g/cm^2^)	0.93 ± 0.15	0.93 ± 0.15	0.93 ± 0.15	0.988
(T-score)^*^	−0.7 ± 1.0	−0.7 ± 1.0	−0.7 ± 1.0	
Other risk factors^*^ in FRAX^®^, ***n***/***N***^@^				
Parent fractured hip (%)	228/2,290 (10.0)	110/1,145 (9.6)	118/1,145 (10.3)	0.577
Previous fracture (%)	117/2,290 (5.1)	55/1,145 (4.8)	62/1,145 (5.4)	0.507
Glucocorticoids (%)	107/2,290 (4.7)	55/1,145 (4.8)	52/1,145 (4.5)	0.767
Rheumatoid arthritis (%)	111/2,290 (4.8)	59/1,145 (5.2)	52/1,145 (4.5)	0.496
Secondary osteoporosis (%)	150/2,290 (6.6)	80/1,145 (7.0)	70/1,145 (6.5)	0.617
Current smoking (%)	372/2,290 (16.2)	191/1,145 (16.7)	181/1,145 (15.8)	0.571
Alcohol 3 or more units/day (%)	115/2,290 (5.0)	52/1,145 (4.5)	63/1,145 (5.5)	0.293

* *Definition same as those of FRAX; BMD, Bone mineral density; T-score reference data, the National Health and Nutrition Examination Survey (NHANES III) for women aged 20–29*.

@ *n, answered yes in the questionnaire; N, total number who answered the questionnaire*.

Univariate analysis showed that the four factors, namely, age, weight, previous fracture history, and height, were the main risk factors for osteoporosis. The index weights of these factors were determined by the multivariate regression and were finally used to score the MOSTAi for each subject. Table 2 shows the regression coefficients for the multivariate and univariate analyses in the development cohort. Finally, through multivariate analysis, only weight, age, and the previous fractures were significant factors in the developmental cohort.

**Table 2:** Regression coefficients for the univariate and multivariable analysis in the developmental cohort.

**Variable**	**Univariate analysis**			**Multivariable analysis**			
	**β**	SE^*^	***p***	**β**	SE^*^	***p***	**Index weight**
Age (vs. 10 years younger)	−0.172	0.030	<0.001	−0.092	0.029	0.001	−1
Body weight (vs. 10 kg lighter)	0.358	0.027	<0.001	0.325	0.030	<0.001	3
Previous fracture (vs. no)	−0.106	0.134	<0.001	−0.086	0.125	0.002	−1
Body height (vs. 10 cm shorter)	0.194	0.047	<0.001	0.022	0.050	0.471	
Parent hip fracture (vs. no)	0.016	0.098	0.579	–	–	–	
Glucocorticoids (vs. no)	−0.010	0.135	0.736				
Rheumatoid arthritis (vs. no)	−0.016	0.130	0.592	–	–	–	
Secondary osteoporosis (vs.no)	−0.021	0.113	0.482	–	–	–	
Smoking (vs. no)	−0.038	0.077	0.202				
Alcohol (vs. no)	0.013	0.138	0.661	–	–	–	

* *SE, standard error*.

**Figure 2:**
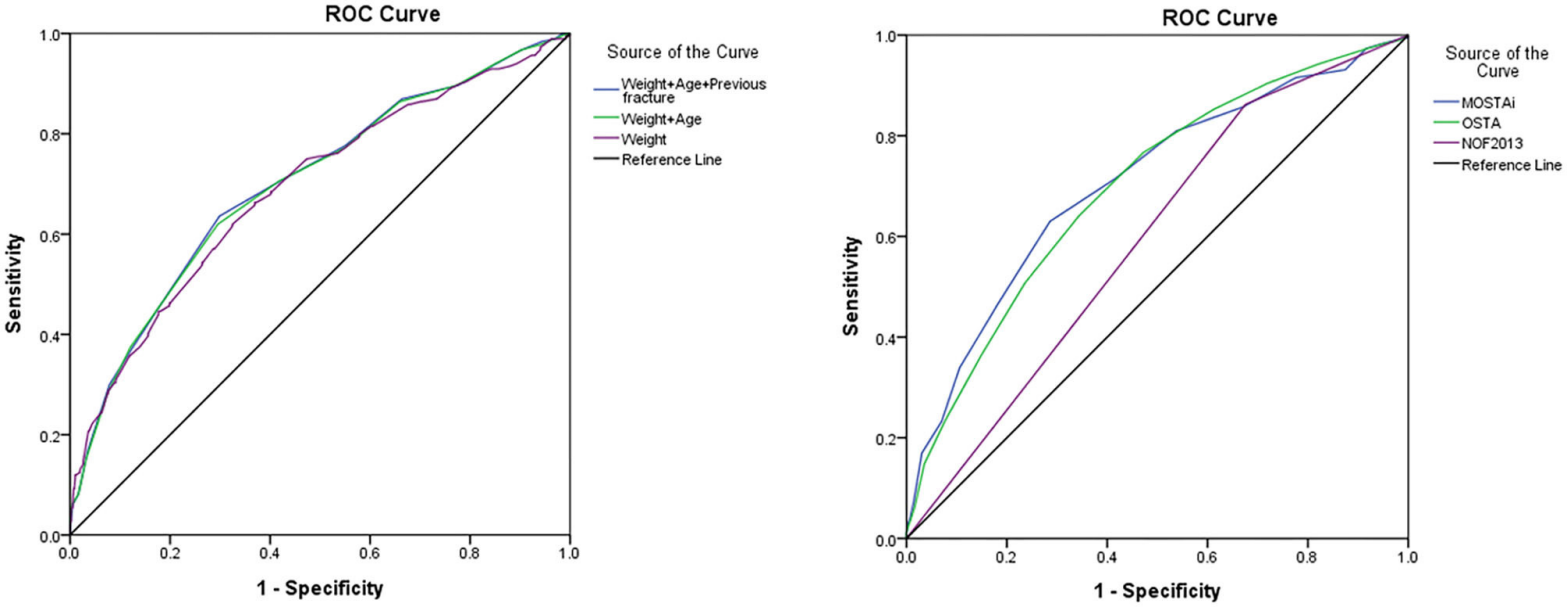
The receiver operating characteristic (ROC) curve of the developmental cohort (left side). The ROC curves for predicting osteoporosis by MOSTAi, OSTA, and NOF 2013 (right side). OSTA, osteoporosis self-assessment tool; NOF 2013, National osteoporosis foundation recommendations in 2013; MOSTAi, modified male osteoporosis self-assessment tool for Taiwan.

The ROC curve for the developmental 1 cohort is shown in Figure 2 (left). The last variables were age, weight, and the previous fractures. A ROC curve analysis was performed to assess the ability of a model to distinguish between the subjects diagnosed with and without osteoporosis. The AUC for weight, weight + age, and weight + age + previous fractures were 0.690 (*p* < 0.001, 95% CI, 0.646–0.734), 0.700 (*p* < 0.001, 95% CI 0.656–0.742), and 0.701 (*p* < 0.001, 95% CI, 0.658–0.744), respectively. The AUC (of the diagnostic tool) <0.7 is considered unacceptable (13). Therefore, the models based solely on the weights were excluded. Besides, the model based only on age and weight behaves almost the same as the model using the three variables. Therefore, for simplicity, we only select age and weight to design the final model. According to the beta coefficient in Table 2, the index weight can be calculated. We converted the regression coefficients of age and weight into units of 10 years and 10 kg, respectively, to simplify the subsequent calculations. Therefore, MOSTAi value could be determined by adding +3 units per 10 kg increase in weight and −1 unit per 10 years increase in age with a reference age of 50 years and weight of 50 kg. The formula for the MOSTAi index is as follows: 0.3 × (weight in kilograms) - 0.1 × (age in years). The MOSTAi index of 1,145 participants in the development cohort was calculated. The average, median, SD, and range of the MOSTAi index were 12.8, 13, 3.4, and 4–23, respectively.

After estimating the ROC curve, the number 11 was selected as the appropriate cut-off value of the MOSTAi index to identify the high-risk subjects diagnosed with osteoporosis in the developmental cohort, with the highest sensitivity (61.96%) and the highest specificity (70.45%). Besides, the positive predictive value (PPV) and negative predictive value (NPV) of MOSTAi in the developmental samples (*n* = 1,145) were 28.64 and 90.63%, respectively. Table 3 shows the comparison between the MOSTAi index, OSTA index, and NOF 2013 in the validation cohort.

**Table 3:** The comparison between modified male osteoporosis self-assessment tool for Taiwan (MOSTAi) index, osteoporosis self-assessment tool (OSTA) index, and National osteoporosis foundation recommendations in 2013 (NOF 2013) in the validation cohort.

**Analysis system**	**Osteoporosis^a^ (–)**	**Osteoporosis^a^ (+)**	***N***
MOSTAi value ≤ 11			
No	683 (0.907^d^, 0.714^e^)	70	753
Yes	273	119 (0.304^b^, 0.630^c^, 0.701^f^)	392
OSTA value ≤ −2			
No	628 (0.902^d^, 0.657^e^)	68	696
Yes	328	121 (0.269^b^, 0.640^c^, 0.654^f^)	449
NOF 2013			
No	310 (0.923^d^, 0.324^e^)	26	336
Yes	646	163 (0.201^b^,0.862^c^,0.413^f^)	809

a *T-score ≤ −2.5 at any site of femoral neck, total hip, or lumbar spine*;

b *Positive predictive value*;

c *Sensitivity*;

d *Negative predictive value*;

e *Specificity*;

f *Accuracy*.

*MOSTAi, Male osteoporosis self-assessment tool for Taiwan; OSTA, Osteoporosis self-assessment tool for Asians; NOF 2013, National osteoporosis foundation recommendations in 2013*.

Using the optimal cutoff value (−2) for 1 OSTA, sensitivity, specificity, PPV, and NPV in the validation cohort were 64.0, 65.7, 26.9, and 90.2%, respectively. Figure 2 (right) shows the ROC curves of NOF 2013, MOSTAi, and OSTA for predicting osteoporosis. The different AUCs of MOSTAi, OSTA, and NOF 2013 were 0.706 (*p* < 0.001, 95% CI: 0.664–0.748), 0.697 (*p* < 0.001, 95% CI: 0.657–0.738), and 0.593 (*p* < 0.001, 95% CI: 0.552–0.634). We conducted a Delong's test between these models. The *p*-value between MOSTAi and OSTA is 0.82, which is not statistically significant. The *p*-value between MOSTAi and NOF 2013 is 0.004, which is statistically significant. In addition, we performed Mcnemar's test for sensitivity and specificity analysis. The *P*-value between MOSTAi and OSTA is <0.0001. In addition, the *P*-value between MOSTAi and NOF 2013 is also <0.0001. Both have reached statistical significance, showing that the sensitivity and specificity of MOSTAi are better.

**Figure 3:**
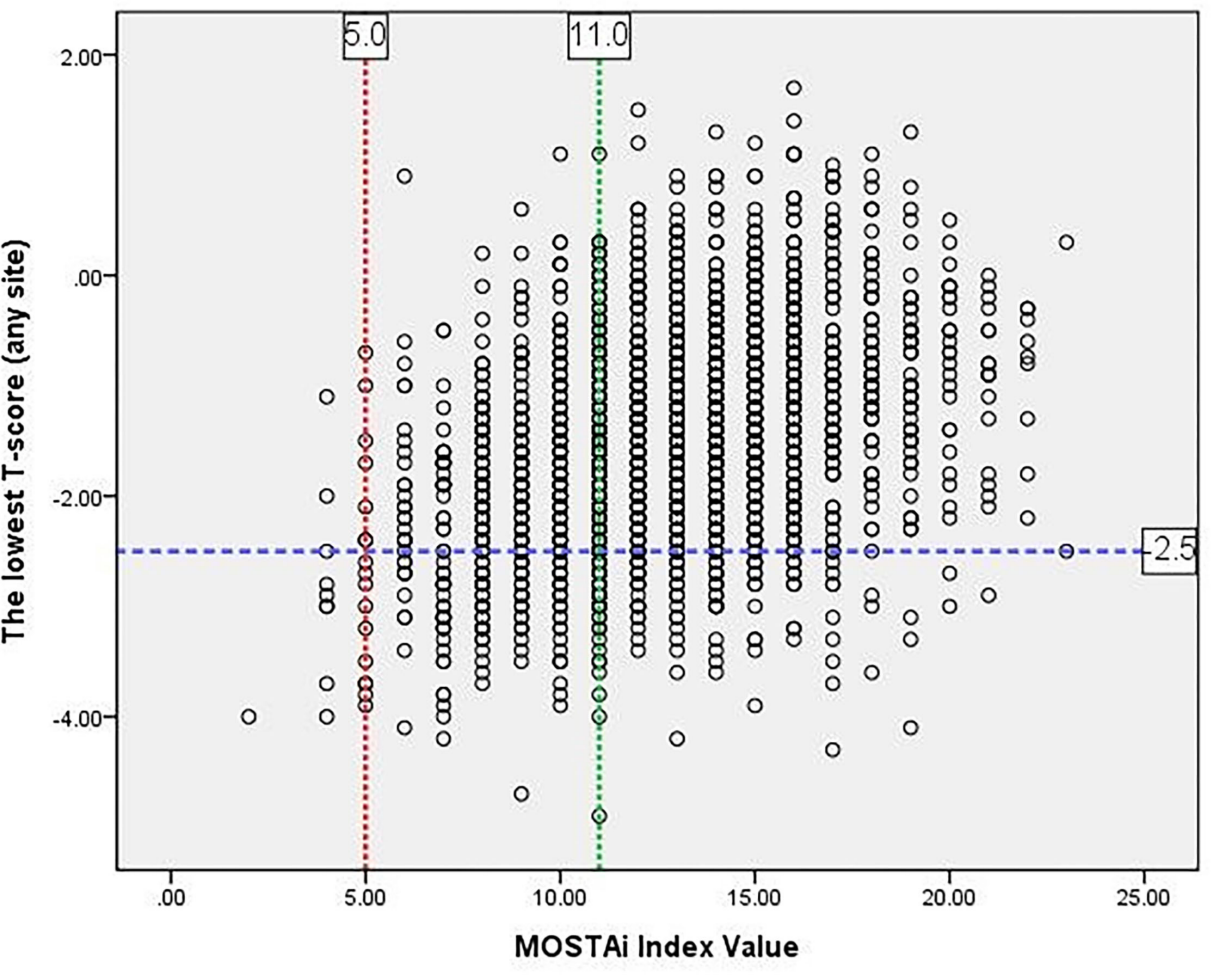
The MOSTAi values vs. lowest *T*-scores at any site for the development sample.

Based on the osteoporosis risk categories used in the Koh study (7), we arbitrarily created three osteoporosis risk categories based on the index. The lowest *T*-scores at any site and the MOSTAi values for the development sample are showed in Figure 3. The high-risk, medium-risk, and low-risk groups included those with MOSTAi index values ≤5, between 5 and 11 (≤11 and >5), and >11.

In the developmental cohort, 65.2, 33.5, and 1.2% of the patients were classified as the low-risk, intermediate-risk, and high-risk groups, respectively. The prevalence of osteoporosis in these groups was 9.4% (70/747), 26.8% (103/384), and 78.6% (11/14). Similarly, in the validation cohort, the patients belonging to the low-risk, medium-risk, and high-risk categories accounted for 65.8, 33.1, and 1.1%, respectively. The prevalence of osteoporosis in these groups was 9.3% (70/753), 29.6% (112/379), and 53.8% (7/13).

## Discussion

Osteoporosis Self-Assessment Tool for Asians was originally developed for the Asian population and later validated in the Caucasian population (14). It has effectively identified postmenopausal women having a risk for osteoporosis. The differences in the accuracy of OSTA are reported in identifying osteoporosis in men as compared 1 with women (15, 16). Lynn et al. observed that the OST index was useful in the population of Caucasian American men when the threshold was ≤ 2 and Hong Kong Chinese men when the threshold was ≤-1 (16). Therefore, the optimal cut-off value for OSTA for predicting osteoporosis in men may vary among the different races. In addition, the different performances of OSTA may be related to how BMD is measured between the surveys (17).

In this study, 373 participants were diagnosed with osteoporosis based on the *T*-score of the femoral neck, lumbar spine, or total hip. Actually, BMD of the femoral neck was used as a measure to define osteoporosis (18). However, the number of patients would have been underestimated if the *T*-score would be measured only on the lumbar spine (*n* = 127), or the femoral neck (*n* = 117), or the total hip (*n* = 7). In addition, it was known that osteoporosis diagnosed at any site could predict the risk of future fracture at other sites (19). According to the previous study, we developed MOSTAi to identify the *T*-score ≤ −2.5 at any site other than just on the femoral neck (20). It does not matter that the tool may be overestimated because the patient still needs DXA to confirm whether he has osteoporosis.

In a previous study of the author, some modifications were made to the original OSTA model by including risk factors from FRAX (21). This led to the development of the Osteoporosis Self-Assessment Tool for Taiwan postmenopausal women (OSTAi). It is a friendly tool for postmenopausal women to self-assess osteoporosis and is commonly used in Taiwan. However, for men, there are currently no self-assessment tools in Taiwan. In this study, the algorithm used to calculate MOSTAi was similar to the original OSTA except that different index weights were considered. In the OSTAi model, the weighted indices of age and weight were −0.2 and +0.2, respectively, and the cut-off value –f −1. In the MOSTAi model, the weighted 1 indices of age and weight were −0.1 and +0.3, respectively. In the MOSTAi formula, the importance of body weight is greater than age, which is different from OSTAi. The reason why lower body weight played a greater role in the development of osteoporosis in the elderly male Taiwanese population is still unclear.

In the validated sample of 1,145 Taiwanese men, the performance of MOSTAi was further compared with OSTA and NOF 2013 (Table 3). The optimal cut-off value of −2 for OSTA resulted in a sensitivity and specificity of 64.0 and 65.7%, respectively, with an AUC of 0.697. An AUC value (of OSTA) <0.7 was considered to be insufficient. MOSTAi showed acceptable sensitivity/specificity (63.0/71.4%) and a high NPV (90.7%). In addition, an AUC (0.706) of MOSTAi in the Taiwanese men was higher than that of OSTA. Therefore, compared with OSTA, the MOSTAi may be a more suitable tool for identifying the Taiwanese men at risk for osteoporosis. Compared with NOF 2013, the accuracy of MOSTAi (70.1%) and AUC (0.706) was improved (41.3% and 0.593, respectively). Although the PPV (30.4) of MOSTAi is not high, it has improved a lot compared with the OSTA (26.9) and NOF 2013 (20.1). Therefore, considering the specificity, sensitivity, and AUC, a MOSTAi is an effective tool for searching men with osteoporosis. It may be due to the formula we designed using local data, so other countries can use the study as a template to set up their own tools to provide better medical services for their population.

The Taiwan OsteoPorosis Survey is the first national survey of osteoporosis in Taiwan that can be used to launch more reliable diagnostic tools. The measurements performed by the same DXA machine and the same technician reduced the inter-modality and inter-operator variations. Although the clinical risk factors in FRAX have been included in MOSTAi, they have not been used to develop OSTA in men. In the near future, different variables can be analyzed for further prospective study design 1 to enhance the predictive power of this tool (22).

In recent years, with the aging of society, male osteoporosis has become an important problem. “The Osteoporotic Fractures in Men (MrOS) Study” focused on osteoporosis and fractures in men aged 65 years and over and published prospective data for more than 16 years (23). It tells us that in the first BMD test, elderly men with a BMD *T* score> −1.50 are highly unlikely to develop osteoporosis during the follow-up period and do not need to undergo another BMD test. The difference between the MOSTAi and MrOS is that MOSTAi provides a simple self-assessment tool that can decide whether to conduct a BMD survey on men without having to conduct a BMD first.

This study has some limitations. First, the population was not randomly selected and the proportion of osteoporosis would have been higher than the general population (24). Some subjects do not participate in the study themselves, but the clinicians may refer certain high-risk patients for osteoporosis assessment, leading to selection bias. However, the prevalence of osteoporosis in Taiwanese men aged 50 years or older (16.3%) was similar to the elderly (17.2%) population (25). Second, although the lower body weight and aging can predict the risk of fractures in the future; further research is needed to check whether MOSTAi can predict this risk in Taiwanese men. On the other hand, although FRAX is most commonly used for predicting osteoporotic fractures, it is still not able to predict patients with the WHO defined osteoporosis. Therefore, we cannot compare MOSTAi with FRAX. Third, although secondary osteoporosis is a clinical risk factor for FRAX, our information about it comes from an item called “secondary osteoporosis” in the questionnaire. So, we do not know the proportion of individuals with these diseases. It is impossible to know their impact on this research.

## Conclusion

A MOSTAi has been proven a simple tool with reasonable 1 sensitivity/specificity, PPV, and a high NPV. Compared with OSTA and NOF 2013, a MOSTAi may be more suitable for identifying the Taiwanese men who are at risk for osteoporosis and further recommending the risk groups for the DXA test.

## Data Availability Statement

The original contributions presented in the study are included in the article/supplementary material, further inquiries can be directed to the corresponding author/s.

## Ethics Statement

This study had been approved by Institutional Review Board of Chang Gung Memorial Hospital (102-1878B). The patients/participants provided their written informed consent to participate in this study. Written informed consent was obtained from the individual(s) for the publication of any potentially identifiable images or data included in this article.

## Author Contributions

D-HL, C-YH, and T-TC contributed to the conception and design of the study. P-CW, Y-CC, Y-YC, J-FC, and S-FY organized the database and performed the statistical analysis. T-TC wrote the first draft of the manuscript. D-HL, C-YH, and T-TC wrote sections of the manuscript. All authors contributed to manuscript revision, read, and approved the submitted version.

## Funding

This work was supported by a grant (CMRPG8K0441) from Chang Gung Memorial Hospital.

## Conflict of Interest

The authors declare that the research was conducted in the absence of any commercial or financial relationships that could be construed as a potential conflict of interest.

## Publisher's Note

All claims expressed in this article are solely those of the authors and do not necessarily represent those of their affiliated organizations, or those of the publisher, the editors and the reviewers. Any product that may be evaluated in this article, or claim that may be made by its manufacturer, is not guaranteed or endorsed by the publisher.
